# High-Density Lipoproteins-Cholesterol (HDL-C) in Women With Gestational Diabetes (GDM): A Predictor for Large Gestational Age (LGA) Babies

**DOI:** 10.7759/cureus.65546

**Published:** 2024-07-27

**Authors:** Alexandre P Pereira, Micaela F Montero, Filipe D Souza, Martha C Jordão, Maria Carolina M Oliveira, Rosiane Mattar, Sergio A Dib, Patricia M Dualib, Bianca de Almeida-Pititto

**Affiliations:** 1 Department of Medicine, Universidade Federal de São Paulo, São Paulo, BRA; 2 Department of Endocrinology, Universidade Federal de São Paulo, São Paulo, BRA; 3 Department of Obstetrics and Gynecology, Universidade Federal de São Paulo, São Paulo, BRA; 4 Department of Preventive Medicine, Universidade Federal de São Paulo, São Paulo, BRA

**Keywords:** maternal lipid, macrosomia, pregestational bmi, high-density lipoprotein (hdl)-cholesterol, large for gestational age (lga), gestational diabetes

## Abstract

Introduction

The present study aimed to evaluate the associations between the clinical and biochemical characteristics of women with gestational diabetes (GDM) and the incidence of large for gestational age (LGA) babies.

Methods

This cohort study included data collected during prenatal follow-up of GDM women from January 2008 to August 2022. Clinical and biochemical variables were compared among small (SGA), adequate (AGA), or large for gestational age (LGA) babies. Associations of the main variables with the incidence of LGA were determined by multiple regression analysis.

Results

Out of 659 women, 56 had LGA, 547 had AGA, and 56 had SGA babies. We observed differences in the means of age, pregestational body mass index (BMI), high-density lipoproteins-cholesterol (HDL-C) levels, gestational weight gain (GWG), and gestational age at birth according to LGA, AGA, and SGA (p < 0.05). All other variables were not different between the groups. The frequencies (%) and relative risk (RR) of LGA babies were evaluated according to HDL-C in the first tertile and/or obesity, with 12.2% and risk ratio (RR)=2.77 (95% confidence interval (CI) 1.35-5.69, p=0.005) if the women had obesity and HDL in the first tertile, 11.3% and RR=2.27 (95% CI 1.03-5.03, p=0.042) if only HDL in the first tertile was present, 10.9% and RR=2.68 (95% CI 1.31-5.48, p=0.007) if the women had only obesity, using as a reference group those women without obesity or HDL-C in the first tertile (4.6% and RR=1) adjusted for age, age at birth and GWG.

Conclusion

In women with GDM, lower levels of HDL-cholesterol during pregnancy, as well as pregestational obesity, seem to be good predictors of the occurrence of LGA babies.

## Introduction

Gestational diabetes mellitus (GDM) is highly prevalent worldwide and has been associated with adverse maternal-fetal outcomes [1]. In the short term, there is a greater risk of preeclampsia, preterm birth, and birth lacerations (for the mother), as well as macrosomia, large for gestational age (LGA) babies, and neonatal hypoglycemia (for the baby) [2]. In the long term, there is an increased risk of developing type 2 diabetes (T2DM) [3] and cardiovascular diseases (for the mother) [4], as well as childhood obesity and diabetes in youth [5]. Therefore, identifying women at greater risk for these adverse outcomes among those with GDM is extremely relevant because it can guide more effective and efficient preventive strategies.

Fetal hyperinsulinemia, stimulated by excess maternal glucose crossing the placental barrier, has an anabolic effect, causing fetuses to become LGA or macrosomia, which in turn is associated with a greater occurrence of complications at birth such as shoulder dystocia; a change in lung maturation; and neonatal hypoglycemia, which can result in brain damage if not correctly managed [1,2].

Along with hyperglycemia during pregnancy, several factors influence birth weight, including prepregnancy body mass index (BMI) and gestational weight gain [6]. Among pregnant women with hyperglycemia, the role of lipid profile and triglyceride (TG) levels during gestation has emerged as potential markers for LGA babies [7].

In this context, it is important to reinforce that index based on TG and high-density lipoproteins-cholesterol (HDL-C), such as the triglyceride/glucose index (TyG) or TG/HDL ratio, have been associated with insulin resistance and cardiometabolic outcomes in non-pregnant adults [8]. The TyG index and the TG/HDL ratio have been shown to be strong predictors of increased cardiovascular risk, with a direct proportional relationship to acute myocardial infarction and stroke [9]. Considering this evidence, TG and correlated indices have gained attention from the scientific community as easily accessible and applicable predictors of adverse health outcomes in clinical practice [10-13]. In pregnant women, the TyG index has been shown to be a predictor of LGA babies [14], and the role of HDL-cholesterol as a marker of a healthy pregnancy is emerging in the literature [15]. However, the role of the lipid profile in pregnant women is still under evaluation [16], and there are few studies on the association of TG and/or HDL-C profiles with maternal-fetal outcomes in women with gestational diabetes, which is a specific population at high risk for adverse fetal outcomes. Considering the potential benefits of the lipid profile in identifying markers of LGA babies in women with gestational diabetes, the current study aimed to assess the associations between infant birth weight (and the occurrence of LGA infants) and TG and HDL-C levels during pregnancy in women with GDM.

## Materials and methods

This was a retrospective cohort study encompassing data collected during the prenatal assistance of 1081 women from the outpatient diabetes and pregnancy service at the Federal University of São Paulo, Brazil, from January 2008 to November 2021.

**Figure 1:**
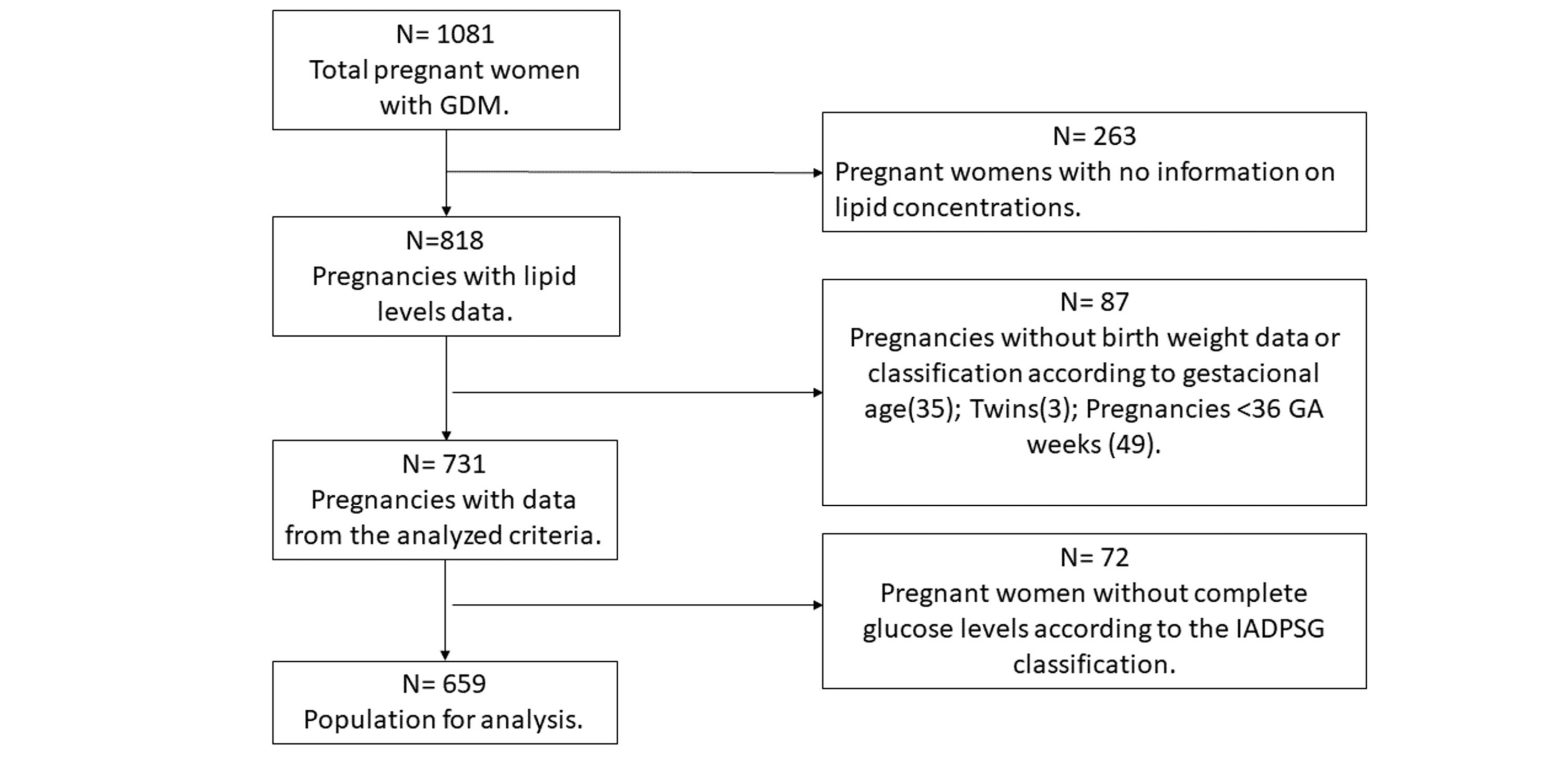
Flowchart of the women with gestational diabetes included in the study GDM: gestational diabetes; IADPSG: International Association of Diabetes and Pregnancy Study Groups; GA: gestational age

All women were above 18 years of age at the time of prenatal care and were diagnosed with GDM during their follow-up, defined by fasting blood glucose ≥ 92 mg/dL and < 126 mg/dL in the first trimester of pregnancy and/or an abnormal 75 g oral glucose tolerance test (OGTT) between 24 and 28 weeks of gestation with fasting blood glucose ≥ 92 mg/dL and/or one-hour ≥ 180 mg/dL and/or two-hour ≥ 153 mg/dL and < 200 mg/dL [17]. For the current study analysis, women who did not have data for the exposure variable (lipid profile) or the outcome variable (birth weight or classification as large for gestational age) were excluded, resulting in a sample of 731 women. From this sample, a further evaluation was conducted, and 72 pregnant women who did not meet the diagnostic criteria described above were excluded, resulting in a final sample of 659 pregnant women (Figure 1).

This project was approved by the Research Ethics Committee of the Paulista School of Medicine, São Paulo Hospital, Federal University of São Paulo (UNIFESP), under protocol number CAAE: 57520822.4.0000.5505.

Variables of interest

Exposure Variables

Lipid profile, mainly TG and HDL-cholesterol. Lipid measurements were taken at the time of GDM diagnosis. The lipid profiles of patients were collected at two time points, either in the first trimester (n=110), or in the second and third trimesters (n=549). TyG (= log [(TG mg/dL x glucose mg/dL)/2]); the BMI was calculated as the mother's prepregnancy weight divided by the square of the mother's height; and HDL-C was stratified into tertiles, defined as follows: T1 < 57 mg/dl; T2 from 57 to 68 mg/dl; and T3 ≥ 69 mg/dl.

Outcome Variable

Infant birth weight and classification of birth weight (small, appropriate, or large for gestational age), according to the INTERGROWTH-21st curve recommended by the WHO [18], which assesses birth weight relative to gestational age at delivery were considered. Small for gestational age (SGA) infants are those below the 10th percentile; appropriate for gestational age (AGA) infants are between the 10th and 90th percentiles; and LGA infants are above the 90th percentile.

Covariates

Prenatal monitoring period: Age; gestational weight gain, blood pressure; fasting blood glucose levels and one-hour and two-hour values for those who underwent the oral glucose tolerance test in the second or third trimester of pregnancy; percentage of insulin use during pregnancy; personal pathological history (comorbidities associated with diabetes and other chronic diseases); obstetric personal history; type of delivery and gestational age at delivery; maternal complications (hypertension, preeclampsia, hypothyroidism); fetal complications (malformations, admission to the intensive care unit, hypoglycemia, hyperbilirubinemia, respiratory distress, neonatal death).

Gestational weight gain was categorized according to the Institute of Medicine (IOM) 2009 values [19], which were defined as inadequate (if under the recommendation), adequate, or excessive weight gain during pregnancy for values below, within, or above the recommended range, respectively. The recommended GWG is 12.5-18 kg, 11.5-16 kg, 7-11.5 kg, and 5-9 kg for women with prepregnancy BMIs classified as underweight (< 18.5 kg/m^2^), normal weight (18.5-24.9 kg/m^2^), overweight (25-29.9 kg/m^2^), and obese (≥ 30 kg/m^2^), respectively.

Statistical analysis

The analysis was conducted using the SPSS for Windows, Version 16.0 (Released 2007; SPSS Inc., Chicago, United States). The sample was stratified into small (SGA), adequate (AGA), and large (LGA) for gestational age. The characteristics of the mothers were compared among these three groups. For continuous variables with a normal distribution, parametric tests were employed (student's t-test or analysis of variance (ANOVA) with Bonferroni correction and Pearson correlation), while nonparametric tests (Mann-Whitney or Kruskal-Wallis and Spearman correlation) were used for other variables with a non-normal distribution. The frequencies of LGA individuals were compared between tertiles of TG and HDL-C. Categorical variables were compared using the chi-square test. Following initial comparative analyses, multiple regression analysis was conducted using the STATA 14 statistical package to obtain the relative risk. The dependent variable was LGA infants, and the variables of interest were HDL-cholesterol tertiles and prepregnancy BMI, with adjustments for covariates. Adjustments were made for all those variables that could interfere with the exposure variable and with the outcome (as a confounding factor) and that were different between the LGA, SGA, or AGA groups. The lipid profiles of patients were collected at two time points, either in the first trimester (n=110), or in the second and third trimesters (n=549). Sensitivity analyses were performed excluding those women who had lipids measured in the first trimester, but the results were not different, and we decided to include all the women in the current analysis. The significance level was set at 5%.

## Results

The analyses were conducted using a database containing information from 659 women with GDM who were followed at the Diabetes Center from January 2008 to November 2021.

A comparative analysis of maternal characteristics contained in this database was performed with the outcome of infant birth weight and its classification (SGA, AGA, or LGA) (Table 1). Regarding pregestational characteristics, Table 1 shows that maternal age at the beginning of pregnancy was lower for mothers who had LGA infants (32.2 (5.2) vs. 34.0 (5.5) vs. 34.7 (4.6) years, p=0.027) than for mothers who had AGA and SGA infants. According to the Bonferroni post-hoc correction, the observed difference was significant between the LGA vs. AGA groups and LGA vs. SGA groups.

**Table 1:** Maternal-fetal characteristics according to SGA, AGA, and LGA babies The values are presented as the mean (standard deviation) or n (percentage). ANOVA was used for continuous variables. Bonferroni correction, if p<0.05: a, vs. SGA; b, vs. AGA. The chi-square test was used for categorical variables. HDL tertile 1: values < 57 mg/dL. Maternal complications: hypertension, preeclampsia, and cesarean delivery. The neonatal complications included hypoglycemia, acute respiratory distress syndrome, prolonged jaundice, malformation, neonatal intensive care unit admission, and neonatal death. The recommended amount of GWG (gestational weight gain) is 12.5-18 kg, 11.5–16 kg, 7–11.5 kg, and 5–9 kg for women with a prepregnancy BMI classified as underweight (< 18.5 kg/m^2^), normal weight (18.5–24.9 kg/m^2^), overweight (25–29.9 kg/m^2^) and obese (≥ 30  kg/m^2^), respectively. SGA: small for gestational age; AGA: appropriate for gestational age; LGA: large for gestational age; BMI: body mass index; OGTT: oral glucose tolerance test

	SGA	AGA	LGA	p
	n=56	n=547	n=56	
Age (years)	34.7 (4.6)	34.0 (5.5)	32.2 (5.2) ^a,b^	0.027
Pregestational BMI (kg/m²)	28.7 (6.3)	29.8 (6.0)	32.1 (6.8)^ a,b^	0.008
Pregestational obesity n (%)	21 (38.9)	233 (44.6)	55 (58.2)	0.017
Parity (2 or more), n (%)	18 (32.1)	196 (35.8)	21 (38.2)	0.964
GDM or previous macrosomia, n (%)	7 (13.2)	58 (11.0)	9 (17.0)	0.411
Smoking, n (%)	13 (23.6)	80 (15.4)	11 (20.0)	0.221
Fasting glucose 1^st^ trimester (mg/dL)	92.4 (10.9)	92.2 (9.0)	93.0 (10.3)	0.908
OGTT 3^rd^ trimester				
Fasting glucose (mg/dL)	92.2 (10.4)	95.9 (10.2)	95.6 (10.6)	0.067
1-hour glucose (mg/dL)	180.1 (29.3)	175.9 (34.0)	174.4 (34.0)	0.729
2-hour glucose (mg/dL)	144.9 (27.4)	145.4 (28.6)	142.6 (31.6)	0.679
Total cholesterol (mg/dL)	229.6 (37.9)	218.7 (46.4)	215.9 (45.0)	0.194
HDL-cholesterol (mg/dL)	70.8 (16.9)	64.2 (14.7) ^a^	59.0 (12.9) ^a,b^	0.001
1 tertiles of HDL-c, n (%)	6 (10.7)	190 (34.7)	26 (46.4)	0.001
LDL- cholesterol (mg/dL)	123.5 (36.9)	119.6 (39.4)	121.0 (40.2)	0.766
Triglycerides (mg/dL)	194.0 (102.6)	183.3 (72.3)	179.96 (66.3)	0.546
Insulin use, n (%)	22 (40)	186 (34.3)	21 (37.5)	0.641
Metformin use, n (%)	0 (0)	9 (6)	1 (6.7)	0.539
Maternal complications, n (%)	35 (63.6)	229 (44.1)	26 (47.3)	0.022
Gestational weigh gain (kg)	8.3 (5.7)	9.2 (6.0)	11.9 (8.0)^ a,b^	0.006
Category of GWG				0.193
Inadequate weight gain, n (%)	15 (28.8)	96 (19.6)	9 (17.6)	
Adequate weight gain, n (%)	19 (36.5)	166 (33.8)	13 (25.5)	
Excessive weight gain, n (%)	18 (34.6)	229 (46.6)	29 (56.6)	
Gestational age at delivery (weeks)	38.0 (1.3)	38.5 (1.2) ^a^	38.5 (1.2)	0.003
Birth weight (kg)	2.5 (0.4)	3.2 (0.3) ^a^	4.0 (0.3)^ a,b^	0.001
Neonatal complications, n (%)	29 (55.8)	188 (36.6)	15 (29.4)	0.011

Mothers who had LGA infants had a greater prepregnancy BMI (32.1 (6.8) vs. 29.8 (6.0) vs. 28.7 (6.3) kg/m², p=0.008) than mothers who had AGA and SGA infants, respectively. According to the Bonferroni correction, the differences were significant between the LGA vs. AGA group and the LGA vs. SGA group (Table 1).

Women who had LGA infants had lower mean values of HDL-cholesterol (59.0 (12.9) vs. 64.2 (14.7) vs. 70.8 (16.9) mg/dL, p=0.001) than mothers who had AGA and SGA infants, respectively. By the Bonferroni method, significant differences were observed among all three groups: LGA vs. AGA, LGA vs. SGA, and AGA vs. SGA. The percentages of women in the first tertile of HDL-C (T1 < 57; T2: 57 to 68; T3 ≥ 69 mg/dL) in the LGA group were (26 (46.4) vs. 190 (34.7) vs. 6 (10.7)%, p=0.001) compared to those in the AGA and SGA groups, respectively (Table 1).

Concerning post gestational characteristics, the means (SD) of gestational weight gain (11.9 (8.0) vs. 9.2 (6.0) vs. 8.3 (5.7) kg, p=0.006) and their categories: inadequate weight (9.0 (17.6) vs. 96 (19.6) vs. 15 (28.8)%, p=0.193), adequate weight (13 (25.5) vs. 166 (33.8) vs. 19 (36.5)%, p=0.193) and excessive weight gain (29 (56.9) vs. 229 (46.6) vs. 18 (34.6)%, p=0.193), gestational age at delivery (38.5 (1.2) vs. 38.5 (1.2) vs. 38.0 (1.3) weeks, p=0.003), birth weight (4.0 (0.3) vs. 3.2 (0.3) vs. 2.5 (0.4) kg, p=0.001), and frequencies of maternal and neonatal complications were significantly different between the LGA, AGA, and SGA groups, respectively (Table 1).

There were no differences between the LGA, AGA, and SGA groups for maternal age, history of GDM or macrosomia in previous pregnancies, percentage of women using insulin or metformin, fasting blood glucose obtained in the first trimester of pregnancy, glucose values from the 75 g OGTT conducted in the second or third trimester of pregnancy, total cholesterol, LDL-cholesterol, and triglycerides.

Table 2 shows the frequencies of LGA babies according to tertiles of the TyG index, triglyceride levels, and HDL-C levels. According to tertiles of TyG and triglycerides, no significant difference was observed in the incidence of LGA. There was a trend toward decreasing LGA incidence across the HDL-C tertiles, as observed in the linear-by-linear analysis (26 (11.7) vs. 16 (7.7) vs. 13 (6.1)%, p=0.035).

**Table 2:** Frequencies of LGA babies according to tertiles of the TyG index, TG levels, and HDL-C levels The values are n (%). Pearson´s chi-square test and linear-by-linear test were performed. TG tertiles: T1 <146; T2:146 to 204; T3 > 204 mg/dl; TyG tertiles: T1 <4.77 T2: 4.77 to 4.93; T3 >4.93; HDL-C tertiles: T1 < 57; T2 57 to 68; T3 ≥ 69 mg/dl. LGA: large for gestational age; TyG: triglyceride/glucose; TG: triglyceride; HDL-C: high-density lipoproteins-cholesterol

	1^st^ Tertile	2^nd^ Tertile	3^rd^ Tertile	p chi-square	p linear-by-linear	
TyG						
LGA n (%)	17 (7.6)	21 (9.6)	18 (8.3)	0.753	0.796	
TG (mg/dl)						
LGA n (%)	21 (9.4)	18 (8.1)	17 (7.9)	0.829	0.570	
HDL-C (mg/dl)						
LGA n (%)	26 (11.7)	16 (7.7)	13 (6.1)	0.095	0.035	

Multiple regression analysis was performed using the incidence of LGA babies as the outcome (dependent variable) and adjusted for age, gestational age at delivery, and gestational weight gain (Table 3). The results showed that women with a BMI ≥ 30 kg/m^2^ or HDL-cholesterol in the first tertile had a greater risk of having an LGA baby than did those in the reference group (women with pregestational BMI < 30 kg/m^2^ and HDL-cholesterol values belonging to tertiles 2 or 3). Compared with women in the reference group, women with pregestational BMI ≥ 30 and HDL-cholesterol levels in tertile 1 also had a greater risk of having an LGA baby. An interaction analysis between the first tertile of HDL-C and BMI ≥ 30 kg/m^2^ was also conducted, and the results did not show any interaction (p=0.130).

**Table 3:** Relative risk (95% CI) between the first tertile of HDL-cholesterol during pregnancy and/or pregestational BMI ≥ 30 kg/m² and the incidence of LGA (large for gestational age) babies Adjustments for age, gestational age at delivery, and gestational weight gain were made. Interaction analysis between HDL-C levels and obesity: p=0.130. HDL-C: high-density lipoproteins-cholesterol; BMI: body mass index; CI: confidence interval

	BMI <30 kg/m^2^	P	BMI ≥ 30 kg/m^2^	p
Tertile 1 of HDL-C	2.27 (1.03 to 5.03)	0.04	2.77 (1.35 to 5.69)	0.05
Tertiles 2 and 3 of HDL-C	1		2.68 (1.31 to 5.48)	0.07

## Discussion

The current study demonstrated a direct association of pregestational BMI and an inverse association of HDL-C with the incidence of LGA infants in women with gestational diabetes, independent of maternal age, gestational weight gain, and gestational age at delivery. Furthermore, no correlation was detected between total cholesterol, LDL-cholesterol, triglyceride, or TyG index levels and the incidence of LGA babies in this sample.

Our analysis of pregestational characteristics according to babies’ birth weight stratified by gestational age revealed an association between elevated pregestational BMI (≥ 30 kg/m²) and the incidence of LGA infants, as expected. Similar findings have been reported in the literature, where women with a higher pregestational BMI (obesity/overweight) at early pregnancy had a greater occurrence of LGA infants [20,21]. In accordance with our findings, these studies suggest that obesity can be considered a significant predictor of LGA babies.

Regarding the lipid profile, we analyzed triglyceride levels and the TyG index in relation to the incidence of LGA in mothers with GDM. We did not observe an association between TG levels, both as a continuous variable and when stratified into tertiles, and the occurrence of LGA babies. The literature reports conflicting results in this regard, and most studies that demonstrate an association between triglycerides and the occurrence of LGA babies do not include or do not separately assess women with GDM [22].

The TyG index, which considers triglyceride and glucose levels, has been suggested to be a potential predictor of insulin resistance, cardiovascular risk, and unfavorable health outcomes [23] in the general population. Lin et al. showed that the TyG index could be a predictor of LGA babies among pregnant women [14], but this study did not enroll pregnant women with GDM and had limitations such as a lack of data regarding gestational weight gain and nutritional status. Our results did not show a correlation between the TyG index and the incidence of LGA babies in mothers with GDM, and we adjusted for gestational weight gain and pregestational BMI. We emphasize that all the pregnant women in our study received the same clinical and nutritional orientation during prenatal assistance during the whole period of study data collection.

Despite the different populations (GDM versus non-GDM women) and adjustments for confounding factors, this lack of association of TG or the TyG index might reflect the clinical profile of women with hyperglycemia during pregnancy. It is worth emphasizing that women with GDM have elevated TG levels because of their insulin resistance profile and already exhibit hyperglycemia [24]. Thus, both TG and glucose levels might not be strong markers for women with GDM since these parameters are quite homogeneous (and elevated) in GDM. Regarding the importance of HDL-cholesterol levels for this population of women with GDM, we also observed that the frequencies of maternal complications were higher in the tertiles with lower HDL levels (Appendix 1).

Additionally, the results did not show a relationship between the incidence of LGA infants and total cholesterol or LDL cholesterol, which is in agreement with other studies regarding lipid profiles and adverse outcomes in pregnant women [16]. However, we did observe that HDL-C levels increased gradually in the LGA, AGA, and SGA infant groups (59.0 (12.9) vs. 64.2 (14.7) vs. 70.8 (16.9) mg/dL, p=0.001), respectively. Similar results were found in other studies [15], showing that maternal total cholesterol or LDL-cholesterol levels were not associated with the incidence of LGA babies, while an inverse association was observed between HDL-cholesterol levels and the incidence of LGA babies [22].

Multivariate regression analysis in the current study confirmed a direct association of pregestational BMI and an inverse association of HDL-C levels with the incidence of LGA babies. The risk of LGA babies was 2.3 to 2.8 times greater in women who were in the lowest HDL-C tertile during pregnancy and/or who had pregestational obesity, respectively, even after adjusting for confounding factors such as age, gestational age at delivery, and gestational weight gain.

An association between pregestational BMI and LGA infants has already been reported [25]. However, data regarding HDL-C are scarce in the literature. A prospective cohort study of 143 pregnant women from the sixth to the 36th week of gestation revealed that in women who were overweight or obese before pregnancy, there was an inverse and statistically significant association between birth weight and HDL-cholesterol, whereas this association was not observed in women with a normal BMI (BMI=20-24.9 kg/m²) [26].

The influence of maternal obesity and lipid profiles on the occurrence of LGA infants has been studied [27-30]. This association is explained by the presence of metabolic factors in maternal obesity, including the lipid profile, which affects the intrauterine environment, fetal growth, and long-term risk of chronic diseases in offspring [27]. Although the exact mechanisms responsible for the effects of maternal obesity on maternal-fetal outcomes are not fully understood, there is clinical and epidemiological evidence indicating that the metabolic profile of these women influences lipid homeostasis and the development of obesity-associated morbidities in offspring [28,29]. Fetal growth in the later weeks of gestation occurs rapidly and is sustained not only by fatty acids passing through the placenta but also by fetal lipogenesis. Fetal hyperinsulinemia in the offspring of mothers with GDM promotes excessive adipose tissue proliferation, resulting in an altered adipokine profile. This increase in body fat in neonates of mothers with GDM is a risk factor for childhood and long-term obesity development [30]. Our results showed that both maternal obesity and low HDL-C influenced the occurrence of LGA babies and did not interact in this population, which could explain why both parameters are relevant for predicting this outcome.

Low HDL-C levels are an important feature of metabolic syndrome and insulin resistance and are therefore frequently present in GDM patients, especially in the latter trimesters of pregnancy [24]. These results reinforce the potential use of low HDL-C as a marker of an unhealthy metabolic profile and undesirable outcomes in pregnant women with GDM. However, to our knowledge, there is a lack of studies investigating the association between HDL-C levels and maternal outcomes.

Another point of concern was the treatment of GDM in terms of glycemic control and insulin use and the potential impact of these factors on the incidence of LGA infants. As shown in Table 1, the percentage of women using insulin did not significantly differ among the LGA, AGA, and SGA groups. It is worth emphasizing that these women received well-supervised prenatal care with a multidisciplinary team and frequent clinic visits, ensuring similar and consistent care and good glycemic control throughout pregnancy.

Regarding the study's limitations, we can highlight the sample size, which may lead to a type II error in statistics, where there is a chance of accepting the null hypothesis (h0) even if it is not true; however, the triglyceride and TyG index values were very similar between groups, which goes against the tendency toward statistical significance if the sample was larger. The lack of some values for the analyzed characteristics in the database, either due to incomplete medical records at the time or due to loss of follow-up of pregnant women, can impact the sample size. Despite these factors, it is relevant to note that triglyceride levels did not differ and that the levels are elevated in women with GDM, emphasizing the importance of HDL in this context. Other potential confounding factors, such as educational level, ethnicity, and smoking status, have been included in the discussion as project limitations as factors that could be used for adjustments. It is important to note it was a public assistance and the women included in the analyses had very similar socioeconomic conditions. With respect to ethnicity, we do not have this information; however, the extensive miscegenation of the Brazilian population is noteworthy. Last, concerning smoking, adjustments were not made because there was no difference in the outcome when comparing the groups (as updated in Table 1). On the other hand, the study has strengths. Data collection was performed during prenatal care, reducing memory and information bias. Additionally, data were consistently added to the database by the same researcher over the years, increasing the reliability of the data. Finally, there is still the opportunity for follow-up of mothers and children to assess the long-term repercussions of these predictive factors.

## Conclusions

Considering the high risk for LGA infants in gestational diabetes, it is relevant to find predictors that could guide preventive strategies. Our findings show that lower HDL-C levels (tertile 1 < 57 mg/dL), as well as pregestational obesity (BMI ≥ 30 kg/m²), might be useful as predictors for the occurrence of LGA infants in mothers with gestational diabetes, independent of maternal age, gestational weight gain, and gestational age at delivery. Furthermore, no correlation was detected between total cholesterol, LDL-cholesterol, triglyceride, or TyG index levels and the incidence of LGA babies in this sample.

It is relevant to notice that several studies have shown that triglyceride levels and associated indices during pregnancy have the potential to predict LGA babies in different populations. However, it is known that women with gestational diabetes have significantly elevated triglyceride levels, making it difficult to use for this prediction. Our results indicate that HDL-cholesterol, along with and independent of elevated BMI, could be a good marker for predicting LGA babies in this population. These predictors can be utilized as low-cost and easily accessible warning signs to guide prevention strategies for the occurrence of LGA infants in women with GDM.

## Appendices

Appendix 1 

**Table 4:** Frequencies of maternal complications according to HDL-cholesterol tertile Values are n (%). Pearson´s chi-square and linear-by-linear test were performed. HDL-C tertiles: T1 < 57; T2 57 to 68; T3 ≥ 69 mg/dl

	1^st^ Tertile	2^nd^ Tertile	3^rd ^Tertile	p chi-square	p linear-by-linear
C-section	142 (63.1)	119 (58.3)	104 (48.8)	0.009	0.003
Hypertensive disease during pregnancy	10 (5.1)	4 (2.1)	3 (1.5)	0.073	0.033
Eclampsia	5(2.6)	3 (1.6)	6 (3.0)	0.638	0.778
